# Coping Styles Among High School Graduates Aiming to Study Medicine in Dealing With Depressive and Anxious Symptoms

**DOI:** 10.3389/fpsyt.2021.735371

**Published:** 2021-11-30

**Authors:** Rebecca Erschens, Teresa Loda, Felicitas Stuber, Anne Herrmann-Werner, Christoph Nikendei, Kaltrina Gashi, Stephan Zipfel, Florian Junne

**Affiliations:** ^1^Department of Psychosomatic Medicine and Psychotherapy, Medical University Hospital Tübingen, Eberhard Karls University Tübingen, Tübingen, Germany; ^2^Competence Centre for University Teaching in Medicine, Baden-Wuerttemberg, Faculty of Medicine Eberhard, Karls University Tübingen, Tübingen, Germany; ^3^Department of General Internal Medicine and Psychosomatics, Heidelberg University Hospital, Heidelberg, Germany; ^4^Department of Psychiatry and Psychotherapy, Saarland University Hospital, Homburg, Germany; ^5^Department of Psychosomatic Medicine and Psychotherapy, Otto von Guericke University Magdeburg, Magdeburg, Germany

**Keywords:** coping styles, high school graduates, medicine, depression, anxiety, medical students, stress and mental well-being

## Abstract

**Background:** Psychological distress, its associated stressors and resilience factors, and the implications derived for the education and training of medical students and physicians have long been the subject of international studies. The study presented here investigated affective symptoms in association with coping styles in the earliest phase of University medical education: high school graduates aiming to study medicine.

**Materials and Methods:** We conducted a self-report survey at a medical school in Germany among high school graduates who indicated being interested in studying medicine at the university's on-campus recruitment day. The questionnaire included validated instruments for the self-assessment of symptoms of depression (i.e., Patient Health Questionnaire; PHQ-9) and anxiety (i.e., Generalized Anxiety Disorder 7 (GAD-7), and participants were also asked to rate functional and dysfunctional behavior-based coping styles for symptoms of depression and anxiety. Additional variables addressed were gender, motivation, interest in studying medicine, and parental employment in medicine.

**Results:** Of 400 high school graduates, 346 (87%) completed the survey. More than 40 (12.5%) and nearly 30 (8.4%) reported relevant symptoms of depression (PHQ-9 sum score ≥10) and anxiety (GAD-7 sum score ≥10), respectively. Among the graduates, young women had higher values for symptoms of depression than young men, and one's interested exclusively in studying human medicine tended to have marginally higher levels of symptoms of depression than ones who were also interested in other subjects. Relevant functional coping styles included seeking social support, relaxing, engaging in sports, listening to or making music, and reading books, whereas relevant dysfunctional coping styles included consuming alcohol, abusing drugs, restrictive eating, watching TV, surfing the Internet, and withdrawing and ruminating.

**Conclusion:** The results clarify the burden and associated resilience factors of premedical high school graduates at the earliest phase of their University education. As such, they reveal ways to address educational and supportive services and support the need for further investigation into factors of success in studying human medicine.

## Introduction

The study of human medicine is undertaken in some of the most sophisticated educational programmes offered by universities. It is characterized by complex courses and intense schedules and workloads, especially before exams, as well as hierarchical structures and exposure to illness and death (1–4). As a consequence, stress, and burnout are prevalent among medical students and physicians, as confirmed by impressive, consistent evidence in international literature (4–6). At a syndrome-based level, there are also significant indications of the high prevalence of symptoms of anxiety and depression (5, 7, 8), the latter of which often occur along with suicidal ideation and thoughts of abandoning medical study (9–11).

Studies have suggested not only that recently enrolled medical students have a significant prevalence of anxiety and depression (12) but also that their first year of study is associated with a particularly high prevalence of depression and anxiety (13–15). Beyond that, Alem et al. (16) have noted a significantly higher incidence of psychological problems among medical students <20 years old and in the preclinical stages of medical training. Kötter et al. (17) have added evidence that students perceive stressors especially intensively in their preclinical years of medical training compared with their clinical years. Considering those trends, Bugaj et al. (2) have emphasized the need for preventive interventions early during medical education.

Among prospective students of medicine in universities, high school graduates aiming to study medicine, or *premedical high school graduates*, continue to be an under-examined group. However, investigating that group of graduates could provide information at the earliest stages of medical school, including about the extent of mental stress, associated private and training-related stressors, and the ability to cope with them. High school graduates interested in studying medicine generally intend to study the subject for many years (18), typically with intrinsic motives, including a particular interest in medicine, personal talents and abilities, the pursuit of personal growth, the expectation of positively impacting their professional and personal development, and the general motivation to help others (19).

Studies examining stress among high school graduates have revealed distress rates ranging from 8% to nearly 30% (20–22). Evidence suggests heterogeneous importance of school- or study-related and private-related stressors, including the school and education system, the grading system, doing homework, and preparing for college, conflicts with parents and pressure from parents to perform and relationships with others [e.g., (21, 23, 24)].

In a cross-sectional study, Erschens et al. (3) investigated the prevalence of stress among l high school graduates aiming to study medicine and their specific private and school-related stressors. They found patterns of stress among the high school graduates similar to patterns among first-year medical students, along with similar rates of general stress (~10%) as measured with the Perceived Stress Questionnaire's subscales of joy, demands, and tension. Added to that, graduates who were particularly interested in medical school demonstrated similar stressors compared with medical students in their third, sixth, and ninth semesters.

However, given the limited or lack of information about specific resilience factors or management strategies among high school graduates aiming to study medicine, this paper aims to answer three questions:

(i) What is the prevalence of symptoms of depression and anxiety among high school graduates?
(ii) What behavior-based styles do high school graduates use to cope with symptoms of depression and anxiety?
(iii) How do high school graduates assess their coping styles regarding their helpfulness (or lack thereof) for coping with symptoms of depression and anxiety?

Various aspects can be regarded as factors potentially related to affective symptoms in the cohort. For one, despite little research on the association between wanting to study medicine and having parents employed in the medical field, it remains open to what extent the desire to study medicine relates to parents' employment in medicine. Parents' expectations for their children to study medicine could impact their distress, while the motivation to study human medicine in itself could be relevant to feelings of depression and anxiety. It is also conceivable that high school graduates who are highly motivated or exclusively interested in studying medicine may anticipate the well-known selectiveness and competitiveness before and during their own medical study. After all, specific selection criteria apply to the study of human medicine (see section Admission Regulations for the Study of Human Medicine in Germany). Another consideration is whether the (tentative) relationship indicating that women report more psychological stress than men also applies to the cohort. Thus, along with investigating affective symptomatology and coping among premedical high school graduates, the study will also investigate possible contributing factors based on the following question:

(iv) What is the association of gender, parents' employment in medicine and motivation to study medicine with symptoms of depression and anxiety among high school graduates?

To our knowledge, no study has examined those questions in the specific population of high school graduates aiming to study medicine.

## Materials and Methods

### Design, Participants, and Procedure

The study was conducted at a medical faculty in Tübingen, Germany, with a paper-and-pencil survey. Prospective participants were high school graduates and individuals interested in pursuing an education in medicine, all of whom were recruited at the university's on-campus recruitment day. Ultimately, 400 high school graduates were invited to participate in the study; no incentives to participate were offered, but they were assured that participation was voluntary and anonymous. At the beginning of the survey, a questionnaire was placed at each desk where a participant was seated. Participants who answered the questionnaire and submitted it to a designated collection site were counted as respondents. Participants who left the questionnaire unanswered, only read the questionnaire out of interest, or abandoned the questionnaire after a few pages were counted as non-respondents. All data were collected before the declaration of the COVID-19 pandemic.

### Admission Regulations for the Study of Human Medicine in Germany

Germany's education system determines the curriculum at schools according to specifications unique to the respective federal state. Two school models, the G8 school model (i.e., A levels after 8 years) and the G9 school model (i.e., A levels after 9 years), can be distinguished. Accordingly, prospective students for medical school are predominantly in the 11, 12, or 13th grade. The general qualification for University entrance (*Abiturprüfung*) includes achievement in core subjects such as German, mathematics, and a foreign language, as well as in other subjects such as linguistics, literature, the arts, the social sciences, mathematics, and the natural sciences.

For years, enrolment in human medicine has significantly outsized the number of places available at state faculties of medicine in Germany, where studying human medicine is incredibly popular and highly regarded (25). In 2019, more than 60,700 high school graduates applied for one of the 11,145 seats for study (i.e., Summer and Winter Semesters combined) at the faculties of medicine, meaning that fewer than one in five was accepted (Hochschulstart).

The study of human medicine entails specific admission restrictions, and the admissions procedure in Germany has recently changed [see (26)]. The allocation of seats for study is divided into the selection procedure of the universities (*Auswahlverfahren der Hochschulen*, 60% of the seats), the so-called “additional suitability rate” (*zusätzlichen Eignungsquote*, 10%), and the best graduation rate (*Abiturbestenquote*, 30%). The wait period will no longer be listed as a criterion beginning in Summer Semester 2022, albeit with some exceptions. The selection procedure of the universities includes grade-dependent and at least two grade-independent criteria, most of which are identical to the criteria of the additional suitability rate. At that stage, grade-independent criteria are considered, including tests of suitability for specialized study, interviews, and oral procedures—for instance, the *Test für Medizinische Studiengänge* and the *Hamburger Naturwissenschaftstest* well as completed professional qualifications and activities, practical and voluntary activities, extracurricular achievements (e.g., awards), and any other qualifications. A detailed overview of Admission Regulations for the Study of Human Medicine in Germany can be found at www.Hochschulstart.de.

### Ethics

The Ethics Committee of the Faculty of Medicine at Tübingen University Hospital approved the study (No. 053/2014BO1), and all participants provided their written informed consent.

### Measurements

The questionnaire sought demographic information such as age, gender, and grade level. It also asked whether the participants' parents worked in the medical field and whether the participants themselves were interested in studying human medicine exclusively or other subjects as well. Symptoms of depression and anxiety were measured using the Patient Health Questionnaire (module PHQ-9 and GAD-7), whereas behavior-based coping styles were evaluated in light of a broad spectrum of newly designed items based on qualitative material, as explained below.

### PHQ-9 and GAD-7

Symptoms of depression were assessed with the PHQ module PHQ-9 (27), which contains nine questions on depression. Responses range from 0 (*not at all*) to 3 (*nearly every day*), such that the PHQ-9 sum score ranges from 0 to 27, with scores of ≥5, ≥10, and ≥15 indicating mild, moderate, and severe symptoms of depression, respectively. The Cronbach's alpha of the PHQ-9 is 0.89 (27, 28). By contrast, the GAD-7 module is a self-report questionnaire for screening generalized anxiety disorder, or GAD. The GAD-7 consists of seven items ranging from 0 (*not at all*) to 3 (*nearly every day*), and sum scores range from 0 to 21, with scores of ≥5, ≥10, and ≥15 indicating mild, moderate, and severe symptoms of anxiety, respectively (29). The Cronbach's alpha of the GAD-7 is also 0.89.

### Coping Styles

Items were developed as a result of focus groups and acording to literature reviews (4, 30) and existing instruments (31, 32). The behavior-based coping styles were grouped as functional coping styles that reduce the risk of developing symptoms of depression and anxiety among high school graduates and vice versa. Both groups of styles have been designed to facilitate the development of specific prevention programmes, improve existing resilient behavior, and to establish programmes to teach the identified styles. The final instrument included 25 functional and dysfunctional coping styles. In a two-tailed design, the high school graduates answered either *yes* (“Yes, I use this coping style) or *no* (“No, I do not use this coping style”) based on whether they had used the coping strategy presented. Second, the graduates rated the styles that they used on a 5-point Likert scale regarding their perceived helpfulness in dealing with symptoms of depression and anxiety (0 = *not at all helpful*, 4 = *completely helpful*). Table 1 shows the list of hypothesized functional and dysfunctional coping styles.

**Table 1:** List of behavior-based functional and dysfunctional coping styles.

**Functional coping styles**	**Dysfunctional coping styles**
Seeking support from friends	Consuming alcohol
Playing sports	Overeating
Relaxing	Abusing stimulant drugs
Reading a book	Surfing in the Internet
Relationship to god or praying	Abusing tranquilliser
Seeking support from classmates	Playing games on the PC or mobile phone
Seeking support from family members	Restrictive eating
Visiting theatre or cultural events	Withdrawing and ruminating
Listening or making music	Smoking cigarettes
Cooking or baking	
Doing active relaxation exercises	
Seeking support from parents	
enjoying nature or going for a walk	
Seeking support from partner	

### Statistical Analysis

#### Descriptive Statistics and Analysis of Symptoms of Depression and Anxiety

Statistical analyses were performed with IBM's SPSS for Windows version 25.0. For descriptive summary statistics, total sum scores, mean values, and standard deviations for the validated PHQ-9 and GAD-7 questionnaires were calculated. Given the sample size, a parametric calculation of group differences was possible, and a normal distribution could be assumed. The sample of high school graduates was assumed to be healthy, and the distribution of data from the PHQ was expected to be naturally “biassed to the left;” hence, a more conservative means of calculation was used, namely via non-parametric tests such as the Kruskal–Wallis test and the Mann–Whitney *U* test, in order to accommodate the psychometric nature of the data. To calculate group differences for gender, parents' medical employment, and a high interest in human medicine, group differences for symptoms of depression and anxiety were analyzed as well. The level of significance for all analyses was set at α = 0.05.

#### Identification of Coping Styles

**Figure 1:**
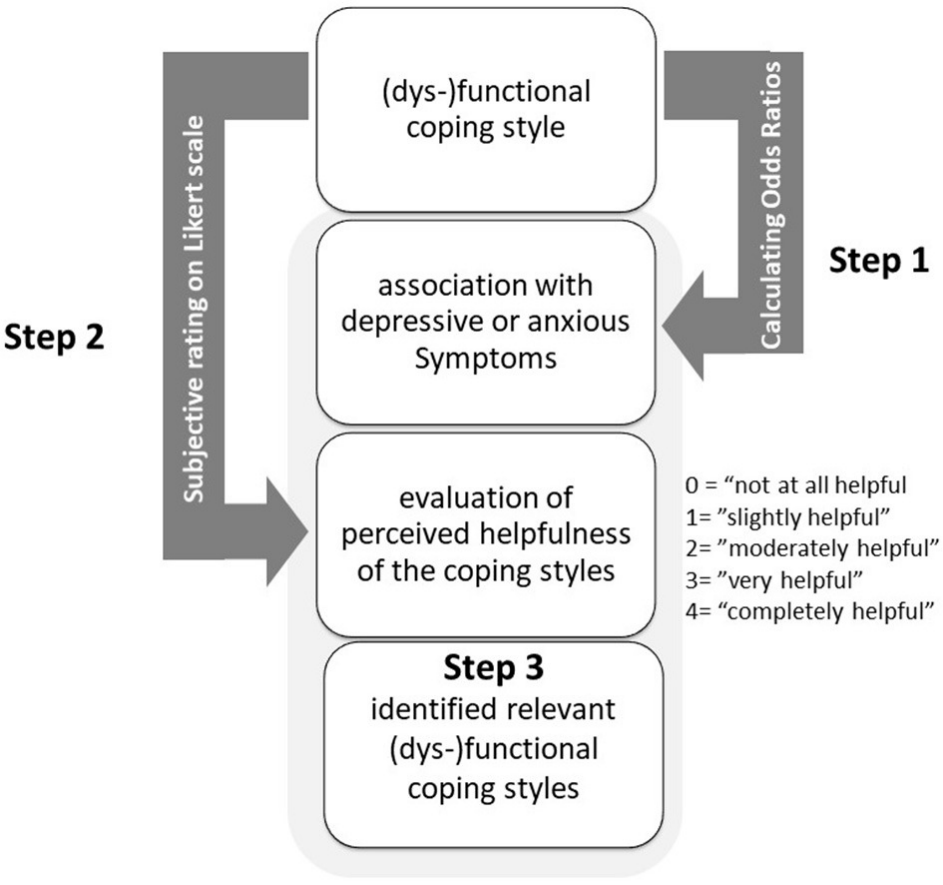
This figure illustrates the three steps to identifying relevant coping styles. First, a correlational association with odd ratios (ORs) between the respective coping strategy and mental distress such as anxiety and depression is established in step 1. Following the objective evaluation, step 2 then includes a subjective assessment to determine whether the respective coping strategy is helpful in association with depressive and anxiety symptoms. In step 3, relevant (dys)-functional coping styles are then defined from the two ratings of coping styles from step 1 and step 2.

Figure 1 illustrates the three steps for identifying relevant coping styles used in the study. First, in Step 1, a correlative relationship with odds ratios (ORs) was established between the respective coping strategy and form of mental distress (i.e., anxiety or depression). To estimate the association between the hypothesized coping styles and symptoms of depression and anxiety, one-way chi-square tests reported as ORs with the corresponding confidence limits and *p* values were calculated. To that purpose, the sample was divided into “depressive or anxious” participants if they showed either relevant symptoms of depression or anxiety (i.e., sum score ≥10) and “hardly depressive or anxious” participants if they showed no relevant symptoms of anxiety or depression (i.e., sum score <10). According to our hypotheses, functional coping styles should be negatively associated with symptoms of depression and anxiety, whereas dysfunctional coping styles should be positively associated with symptoms of depression and anxiety. The ORs mean that when using a functional coping strategy, the probability of not having significant symptoms of depression and anxiety is higher than when not using such a strategy. Vice versa, when using a dysfunctional coping strategy, the probability of having significant symptoms of depression and anxiety is higher than when not using such a strategy. Step 2 involved a subjective assessment to determine whether the respective coping strategy was helpful in handling symptoms of depression and/or anxiety. The high school graduates rated the styles that they used on a 5-point Likert scale regarding their perceived helpfulness in dealing with symptoms of depression and anxiety (0 = *not at all helpful*, 4 = *completely helpful*). For the subjective evaluation, in addition to mean values (*M*), the median value (*Med*), modal value (*Mod*), and percentage rate of the perceived helpfulness of the strategy were included. Last, in Step 3, relevant (i.e., dysfunctional) coping styles were identified from the two coping style assessments in Steps 1 and 2.

Using an exploratory binary logical model, Erschens et al. (30) tested the coping styles illustrated in Table 1 for their relevance and association with stress and burnout among medical students in different stages of their University education.

## Results

### Response Rate and Sample Description

Table 2 provides an overview of the characteristics of the study population. Of 400 high school graduates recruited, 346 (86.5%) participated in the study. Most participants were young women (*n* = 254, 73.4%), and participants were from 15 to 20 years old (*M* = 17.06, *SD* = 1.85). All 346 participants were interested in studying human medicine, although nearly 70% were exclusively interested in human medicine. Most of the graduates (65%) reported to graduate from school in 2 years.

**Table 2:** Characteristics of the study population and other variables.

**Variable (*n* = total number of responses)**		***n*/*N*; %**
Response rate		*N* = 346/400; % = 86.5
Gender (*n =* 345)	Female	*n* = 254; % = 73.4
	Male	*n* = 92; % = 26.6
Age (*n =* 343)	M; Sd	17.06; 1.85
	Range	15–20
Years to graduate from	2 years	*n =* 223; % = 64.4
high school (*n =* 346)	1 year:	*n =* 123; % = 35.6
Parental medical	Yes	*n =* 81; % = 23.5
employment (*n =* 345)	No	*n =* 264; % = 76.5
Interests in	Interests in human medicine:	*n =* 346; % = 100
	Interests in molecular medicine:	*n =* 47; % = 13.6
	Interests medicine technics	*n =* 26; % = 7.5
	Interests in neuroscience	*n =* 68; % = 19.7
Only interests in human	Yes	*n =* 241; % = 69.9
medicine (*n =* 345)	No	*n =* 104; % = 30.1

### Symptoms of Anxiety and Depression

This section answers the following question:

(i) What are the prevalence rates of symptoms of depression and anxiety among high school graduates aiming to study medicine?
(iv) What are the associations of gender, parents' medical employment, and motivation to study medicine with symptoms of depression and anxiety among high school graduates?

Table 3 presents results for symptoms of depression and anxiety overall and in association with other variables according to statistics calculated with the Mann–Whitney *U* test and psychometric properties. The respondents showed mean values of *M* = 5.50 for symptoms of depression and *M* = 4.99 for symptoms of anxiety. According to PHQ-9 and GAD-7 scores, 43 (12.5%) participants reported relevant symptoms of depression and 29 (8.4%) participants reported relevant symptoms of anxiety. More than 5% of the graduates (*n* = 19) reported relevant symptoms of depression as well as anxiety. Analysis of the effects of the variables (i.e., Question iv) with the Mann–Whitney *U* test revealed a significant effect of gender with *U* = 9392, *Z* = −2.662, *p* = 0.008^**^. Young women in the sample showed higher values for symptoms of depression (*M* = 5.75, *SD* = 3.93) than young men (*M* = 4.80, *SD* = 4.02). Graduates who were solely interested in studying medicine tended to have a marginally higher level of symptoms of depression (*M* = 5.82, *SD* = 4.23) than students who were interested in other subjects as well (*M* = 4.76, *SD* = 3.22) and *U* = 10,872, *Z* = −1.853, *p* = 0.064. No differences in depressive or anxious symptoms surfaced between high school graduates whose parents were employed in a medical profession vs. ones whose parents were not.

**Table 3:** Symptoms of depression and anxiety in association to the three additional variables.

			**Variables^a^**		
	**Dimensions/Test**	**Total value**	**Gender**	**Interested only in human medicine**	**Parents work in medical field**
Depressive symptoms (PHQ-9)	M (SD)	*M =* 5.50 (3.97)	♀: *M =* 5.75 (3.93)	Yes: *M =* 5.82 (4.23)	Yes: *M =* 5.47 (4.72)
			♂: *M =* 4.80 (4.02)	No: *M =* 4.76 (3.22)	No: *M =* 5.51 (3.72)
Relevant depressive symptom	PHQ sum score ≥10	*N =* 43; 12.5%	♀: *n =* 33; 13.0%	Yes: *n =* 7; 6.7%	Yes: *n =* 8; 9.9%
			♂: *n =* 10; 10.9%	No: *n =* 36; 14.9%	No: *n =* 35; 13.3%
	Test statistic^b^		*U* = 9,392, *Z* = −2.662, *p* = 0.008^**^	*U* = 10,872, *Z* = −1.853, *p* = 0.064^¥^	*U* = 9,946, *Z* = −0.743, *p* = 0.457
Anxious symptoms (GAD-7)	M(SD)	*M =* 4.99 (3.36)	♀: *M =* 5.04 (3.23)	Yes: *M =* 5.22 (3.57)	Yes: *M =* 5.16 (3.90)
			♂: *M =* 4.87 (3.72)	No: *M =* 4.47 (2.78)	No: *M =* 4.94 (3.17)
Relevant anxious symptoms	GAD sum score ≥10	*N =* 29; 8.4%	♀: *n =* 20; 7.9%	Yes: *n =* 5; 4.8%	Yes: *n =* 7; 8.6%
			♂: *n =* 9; 9.8%	No: *n =* 24; 10.0%	No: *n =* 22; 8.3%
	Test Statistic^a^		*U* = 10,532, *Z* = −1.162, *p* = 0.245	*U* = 11,055, *Z* = −1.853, *p* = 0.118	*U* = 10,516, *Z* = −0.123, *p* = 0.902

a *Analysis of the variables from question (iv)*;

b *Respective Test Statistic with Man-Whitney-U and psychometric properties*.

¥ *Marginal significant*.

** *Highly significant*.

Table 4 shows the contingencies with intersections for symptoms of depression and anxiety in a four-field table. In the sample, 289 (84.5%) of the high school graduates reported no relevant symptoms of anxiety or depression (i.e., PHQ score <10), whereas 24 (7.0%) reported relevant depressive symptomatology (i.e., PHQ-9 score ≥10) but no relevant anxious symptomology (i.e., GAD-7 score <10) and 10 (2.9%) reported relevant anxious symptomology (i.e., GAD-7 score ≥10) but no relevant depressive symptomatology (i.e., PHQ-9 score <10). The other 19 (5.6%) reported both relevant depressive and anxious symptomatology. The correlation of depression to anxiety (*R*^2^ = 0.589) according to Cohen (33) was rated as being “high.”

**Table 4:** Table of contingents with the intersections for depressive and anxious symptoms.

	**PHQ-9**		
**GAD-7**	**No to less depressive symptoms (Score 0–9)**	**Relevant depressive symptoms (Score ≥10)**	**Sum**
No to less anxious symptoms	*n =* 289	*n =* 24	*n =* 313
(Score 0–9)	84.5%	7.0%	91.5%
Relevant anxious Symptoms	*n =* 10	*n =* 19	*n =* 29
(Score ≥10)	2.9%	5.6%	8.5%
Sum	*n =* 299	*n =* 43	*n =* 342
	87.4%	12.6%	100%
Correlation			***R***^**2**^ = 0.589^*a*^

a *Spearman-Rho-correlation*.

Table 5 presents a psychometric overview of the reported symptom burden of feelings of depression and anxiety for the individual items on the PHQ-9 and GAD-7 ordered by their summed value—that is, the sum of the individual response loadings. The top five reported symptoms of depression were tiredness and fatigue (sum score = 385), loss of appetite or increased feelings of hunger (283), sleep problems (271), loss of interest (229), and feelings of sadness (209). Eight of the high school graduates (2.3%) reported having thoughts of life fatigue nearly every day. Symptoms of anxiety were specifically characterized by five symptoms: worrying about various things (sum score = 333), irritability (331), nervousness (319), problems with relaxing (226), and feeling unable to stop worrying (221).

**Table 5:** Symptoms of depression and anxiety in total and in association to the three additional variables.

**Item number^a^**	**PHQ 9 Depression**					
	**Item**	**Response category (score)**				
		**Not at all (0)**	**Several days (1)**	**More than half a days (2)**	**Nearly every day (3)**	**Sum^b^**
4	Feeling tired or having little energy	18.3%, *n =* 63	58.3%, *n =* 201	17.1%, *n =* 59	6.4%, *n =* 22	385
5	Poor appetite or overeating	44.6%, *n =* 154	34.2%, *n =* 118	15.7%, *n =* 54	5.5%, *n =* 19	283
3	Trouble falling or staying asleep, or sleeping too much	48.4%, *n =* 167	32.5%, *n =* 112	11.3%, *n =* 39	7.8%, *n =* 27	271
1	Little interest or pleasure in doing things	41.4%, *n =* 143	51.6%, *n =* 178	6.1%, *n =* 21	0.9%, *n =* 3	229
2	Feeling down, depressed, or hopeless	52.5%, *n =* 181	36.8%, *n =* 127	8.4%, *n =* 29	2.3%, *n =* 8	209
6	Feeling bad about yourself—or that you are a failure or have let yourself or your family down	58.3%, *n =* 201	32.2%, *n =* 111	6.1%, *n =* 21	3.5%, *n =* 12	189
7	Trouble concentrating on things, such as reading the newspaper or watching television	56.3%, *n =* 193	36.4%, *n =* 125	5.2%, *n =* 18	2.0%, *n =* 7	182
8	Moving or speaking so slowly that other people could have noticed? Or the opposite—being so fidgety or restless that you have been moving around a lot more than usual	76.2%, *n =* 263	19.7%, *n =* 68	3.2%, *n =* 11	0.9%, *n =* 3	99
9	Thoughts that you would be better off dead or of hurting yourself in some way	91.3%, *n =* 315	5.5%, *n =* 19	0.9%, *n =* 3	2.3%, *n =* 8	49
**Item number**	**Generalized anxiety disorder 7-item scale**					
	**Item**	**Response category (score)**				
		**Not at all (0)**	**Several days (1)**	**More than half a days (2)**	**Nearly every day (3)**	**Sum^a^**
3	Worrying too much about different things	28.1%, *n =* 97	51.9%, *n =* 179	15.4%, *n =* 53	4.6%, *n =* 16	333
6	Becoming easily annoyed or irritable	25.5%, *n =* 88	57.1%, *n =* 197	13.3%, *n =* 46	4.1%, *n =* 14	331
1	Feeling nervous, anxious, or on edge	22.9%, *n =* 79	64.9%, *n =* 224	9.0%, *n =* 31	3.2%, *n =* 11	319
4	Trouble relaxing	48.7%, *n =* 167	40.2%, *n =* 138	7.6%, *n =* 26	3.5%, *n =* 12	226
2	Not being able to stop or control worrying	50.1%, *n =* 173	38.3%, *n =* 132	9.0%, *n =* 31	2.6%, *n =* 9	221
5	Being so restless that it's hard to sit still	65.5%, *n =* 226	26.4%, *n =* 91	4.6%, *n =* 16	3.5%, *n =* 12	159
7	Feeling afraid as if something awful might happen	71.9%, *n =* 248	20.9%, *n =* 72	4.9%, *n =* 17	2.3%, *n =* 8	130

a *Original item numbering from the original instruments. The authors ranked the items according to their importance for the symptoms (in accordance to sum score)*.

b The sum score is made up of the sum of the individual response loads (x). For example, the sum score = 385 for the item “Feeling tired or having little energy” is made up of n = 63*(0) + n = 201*(1) + n = 59*(2) + n = 22*(3).

### Functional Coping and Dysfunctional Styles Among the High School Graduates

This section analyses the following questions:

(ii) What behavior-based styles do high school graduates use to cope with symptoms of depression and anxiety?
(iii) How do high school graduates assess their coping styles in respect to their helpfulness (or lack thereof) for coping with symptoms of depression and anxiety?

Table 6 reports the identified coping styles within the three-step calculation (see Figure 1). In particular, it details how often each coping strategy is used (“Total use”), its respective OR, and its subjective evaluation. In addition to the mean value the modal value and the percentage of the perceived helpfulness of a particular strategy in dealing with feelings of depression and anxiety are also presented. The division into functional and dysfunctional coping styles hypothesized in Table 1 was confirmed by statistics, and the following section reports the styles ultimately included in the overall assessment as so-called “Relevant coping styles.”

**Table 6a:** Reporting on identified functional coping strategies with associated OR, frequencies of use, and subjective assessment of the usefulness of the strategy.

**Probability of depressive and anxious symptoms**								**Total use^a^**	**Evaluation of perceived helpfulness^b^**
**Functional coping style**	**No**		**Yes**		**χ^**2**^**	***p***	**OR (95% CI)**	***N*, %**	***M* (Sd); Med Mod; %**
	***n***	**%**	***n***	**%**					
1. Support from family members	260	87.5	37	12.5	26.59	<0.01^**^	5.21 (2.66–10.20)	*N =* 297, 86.3%	*M =* 2.92 (1.39); Med = 3.00; Mod = 4
No use	27	57.4	20	42.6			1		*Very helpful up to completely helpful: 74.2%* *moderately helpful: 8.9%* *Slightly helpful up to not at all helpful: 16.9%*
2. Support from friends	269	85.7	45	14.3	13.05	<0.01^**^	3.99 (1.80–8.83)	*N =* 314, 91.3%	*M =* 2.72 (1.13); Med = 3.00; Mod = 3
No use	18	60.0	12	40.0			1		*Very helpful up to completely helpful: 66.7%* *moderately helpful: 22.5%* *Slightly helpful up to not at all helpful: 10.8%*
3. Relaxing	232	88.2	31	11.8	18.48	<0.01^**^	3.54 (1.95–6.44)	*N =* 263, 76.5%	*M =* 2.45 (1.52); Med = 3.00; Mod = 4
No use	55	67.9	26	32.1			1		*Very helpful up to completely helpful: 60.2%* *moderately helpful: 14.8%* *Slightly helpful up to not at all helpful: 25%*
4. Playing sports	219	85.5	37	14.5	3.24	0.072	1.74 (0.95–3.20)	*N =* 256, 74.4%	*M =* 2.51 (1.63); Med = 3.00; Mod = 4
No use	68	77.3	20	22.7			1		*Very helpful up to completely helpful: 64.9%* *moderately helpful: 7.9%* *Slightly helpful up to not at all helpful: 27.2%*
5. Reading a book	195	87.1	29	12.9	6.10	<0.05^*^	2.05 (1.15–3.64)	*N =* 224, 65.1%	*M =* 1.87 (1.56); Med = 2.00; Mod = 0
No use	92	76.7	28	23.3			1		*Very helpful up to completely helpful: 42.6%* *moderately helpful: 17.7%* *Slightly helpful up to not at all helpful: 39.7%*
6. Support from classmates	198	87.2	29	12.8	6.20	<0.05^*^	2.07 (1.16–3.70)	*N =* 227, 66.2%	*M =* 1.78 (1.42); Med = 2.00; Mod = 0
No use	89	76.7	27	23.3			1		*Very helpful up to completely helpful: 38.3%* *moderately helpful: 25.5%* *Slightly helpful up to not at all helpful: 36.2%*
7. Listening or making music	257	83.4	51	16.6	0.000	0.99	1.01 (0.40–2.55)	*N =* 308, 89.5%	*M =* 2.85 (1.27); Med = 3.00; Mod = 4
No use	30	83.3	6	16.7			1		*Very helpful up to completely helpful: 70.6%* *moderately helpful: 15.7%* *Slightly helpful up to not at all helpful: 13.7%*

a *This N indicates the number of respondents stating that they use these coping strategies to deal with depressive and anxious feelings. Multiple responses were possible*.

b *The right column indicates the subjective evaluation of the individual coping strategies. Therefore, the mean value, modal value and median were presented. In addition, the percentage of usefulness of each strategy in coping with depressive and anxious feelings was calculated*.

* *Significant*.

** *Highly significant*.

**Table 6b:** Reporting on identified dysfunctional coping strategies with associated OR, frequencies of use, and subjective assessment of the usefulness of the strategy.

**Probability of depressive and anxious symptoms**								**Total use^a^**	**Evaluation of perceived helpfulness^b^**
**Dysfunctional coping style**	**Yes**		**No**		**χ^**2**^**	***p***	**OR (95% CI)**	***N*, %**	***M* (Sd); Med Mod; %**
	***n***	**%**	***n***	**%**					
1. Drinking alcohol	19	44.2	24	55.8	22.77	<0.01^**^	4.75(2.40–9.40)	*N =* 43, 12.5%	*M =* 0.17 (0.66); Med = 0.00; Mod = 0
No use	43	14.3	258	85.7			1		*Slightly helpful up to not at all helpful: 93.9%* *Moderately helpful: 3.2%* *Very helpful up to completely helpful: 2.9%*
2. Taking stimulant drugs	7	38.9	11	61.1	5.60	<0.05^*^	3.14 (1.16–8.45)	*N =* 18, 5.2%	*M =* 0.11 (0.62); Med = 0.00; Mod = 0
No use	55	16.9	271	83.1			1		*Slightly helpful up to not at all helpful: 97.1%* *Moderately helpful:0.3%* *Very helpful up to completely helpful: 2.6%*
3. Surfing in the Internet	44	24.2	138	75.8	9.90	<0.01^**^	2.55 (1.41–4.63)	*N =* 182, 52.9%	*M =* 1.03 (1.23); Med = 0.00; Mod = 0
No use	18	11.1	144	88.9			1		*Slightly helpful up to not at all helpful: 63.6%* *Moderately helpful: 22.3%* *Very helpful up to completely helpful: 14.1%*
4. Restrictive Eating	24	29.6	57	70.4	9.66	<0.01^**^	2.49 (1.39–4.49)	*N =* 81, 23.5%	*M =* 0.26 (0.80); Med = 0.00; Mod = 0
No use	38	14.4	225	85.6			1		*Slightly helpful up to not at all helpful: 92.7%* *Moderately helpful: 3.5%* *Very helpful up to completely helpful: 3.8%*
5. Watching TV	27	27.8	70	72.2	8.80	<0.01^**^	2.34 (1.32–4.13)	*N =* 97, 28.2%	*M =* 0.46 (1.00); Med = 0.00; Mod = 0
No use	35	14.2	212	85.8			1		*Slightly helpful up to not at all helpful: 84.5%* *moderately helpful: 8.5%* *Very helpful up to completely helpful: 7.0%*
6. Withdrawing and ruminating	45	22.8	152	77.2	7.10	<0.01^**^	2.25 (1.23–4.12)	*N =* 197, 57.4%	*M =* 0.99 (1.14); Med = 0.50; Mod = 0
No use	17	11.6	129	88.4			1		*Slightly helpful up to not at all helpful: 65.2%* *Moderately helpful: 22.5%* *Very helpful up to completely helpful: 12.3%*

a *This N indicates the number of respondents stating that they use these coping strategies to deal with depressive and anxious feelings. Multiple responses were possible*.

b *The right column indicates the subjective evaluation of the individual coping strategies. Therefore, the mean value, modal value and median were presented. In addition, the percentage of usefulness of each strategy in coping with depressive and anxious feelings was calculated*.

* *Significant*.

** *Highly significant*.

Based on the overall assessment, seven relevant functional coping styles were identified: getting social support from family, getting social support from friends, getting social support from classmates, relaxing, exercising, reading books, and making, or listening to music. Differences emerged between the mathematical importance of a strategy and its association with depressive and anxious experiences and subjective ratings of the helpfulness of coping styles. Social support from family (OR = 5.21), friends (OR = 3.99), and classmates (OR = 2.07), as well as relaxing (OR = 3.54) and reading books (OR = 2.05), were significantly associated with reporting fewer or no relevant symptoms of depression or anxiety with their respective ORs. For example, an OR of 5.21 for the coping strategy “Seeking support from family” means that if a high school graduate “Requests their family for help,” then the OR is 5.21 higher, thereby showing no more relevant symptoms of anxiety or depression than if the graduate did not use the strategy. In the final column in Table 6, respondents' subjective ratings of each coping strategy is presented. The coping styles of “Playing sports” and “Listening to or making music” provided only marginally significant or non-significant ORs, respectively. In the subjective assessments of the high school graduates, however, these coping styles remained helpful in managing symptoms of depression and anxiety. Nearly 65% of graduates experienced sports as being very helpful or completely helpful, while more than 70% rated listening to or making music as very helpful.

The six relevant dysfunctional coping styles identified among respondents were consuming alcohol (OR = 4.75), abusing drugs (OR = 3.14), restricting their eating (OR = 2.49), watching television (OR = 2.34), surfing the Internet (OR = 2.55), and withdrawing or ruminating (OR = 2.25). The mathematical significance of a dysfunctional coping strategy and its association with symptoms of depression and anxiety and the graduates' subjective ratings of coping styles were consistent. For example, an OR of 4.75 for the coping strategy “Drink more alcohol” implies that if a high school graduate consumes more alcohol, then the OR is 4.75 higher for the tendency to report more relevant symptoms of anxiety or depression than if the graduate does not use that strategy. The most frequently used dysfunctional coping styles included withdrawing and ruminating (*n* = 197, 57.4%), followed by surfing the Internet (*n* = 182, 52.9%), restrictive eating (*n* = 81, 23.5%), consuming alcohol (*n* = 43, 12.5%), and abusing drugs (*n* = 18, 5.2%), all of which had the highest ORs but were used less frequently. Consuming alcohol, restrictive eating, and abusing drugs were also subjectively rated by 90% of graduates as being slightly helpful or not at all helpful for coping with symptoms of depression and anxiety.

**Figure 2:**
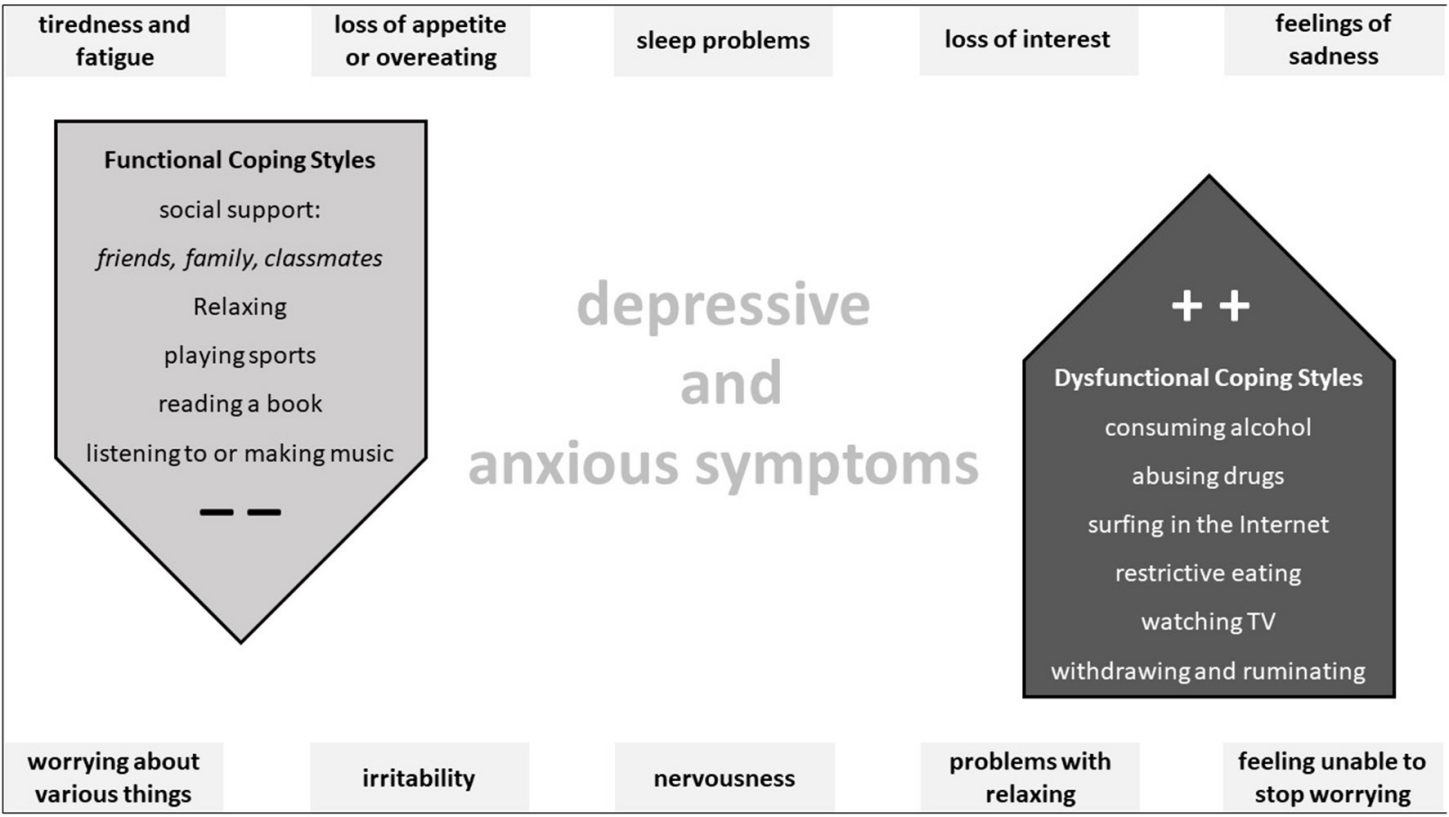
This figure shows the main functional and dysfunctional coping styles in the middle and the main symptoms characterizing depressive and anxious distress in high school graduates around the chart.

Figure 2 illustrates the most important functional and dysfunctional coping styles as well as the major symptoms that characterize the burden of depression and anxiety among the high school graduates.

## Discussion

The study presented herein was conducted to answer four questions:

(i) What are the prevalence rates of symptoms of depression and anxiety among high school graduates aiming to study medicine?
(ii) What coping styles do high school graduates use to address symptoms of depression and anxiety?
(iii) How do high school graduates assess coping styles regarding their helpfulness or lack thereof in dealing with symptoms of depression and anxiety?
(iv) What is the association of gender, parents' medical employment, and motivation to study medicine with symptoms of depression and anxiety among high school graduates?

In the following sections, the results of the study are summarized under the heading of the respective sub-question and compared with the findings in the literature. After that, the methodological limitations are discussed, followed by a conclusion with direct implications and perspectives for future studies.

### Depression and Anxiety Among High School Graduates: Questions I and IV

High school graduates had mean scores of 5.50 for symptoms of depression and of 4.99 for symptoms of anxiety. In particular, 43 (12.5%) and 29 (8.4%) graduates suffered from clinically relevant symptoms of depression and anxiety, respectively (cumulative score ≥10), according to the PHQ. More than 5% (*n* = 19) of the graduates suffered from clinically relevant symptoms of depression and anxiety concurrently. Their symptomatology was characterized by fatigue and exhaustion, a loss of appetite or increased feeling of hunger, trouble sleeping, a loss of interest, a feeling of sadness, worry about various things, irritability, nervousness, problems with relaxing, and rumination.

The literature contains limited information about the population examined in the study, both in general and in relation to affective symptoms. Diab et al. (24) found (severe) affective symptoms ranging from 14 to 20% in young high school graduates who had already been accepted to medical school. Other studies found relevant affective prevalence rates ranging from 20 to 50% among high school graduates (23, 34), whereas Wege et al. (12) found a prevalence of symptoms of major depression or anxiety of ~5% among recently enrolled medical students in Germany.

The results regarding premedical high school graduates reported here align with the heterogeneity of results in research on stress, burnout, and affective symptoms among medical students at different stages of their University education. There are indications that distress is high at the beginning of medical school and either subsequently levels off or continues to increase (4, 5, 7). Rotenstein et al. (5) who quantified symptoms of depression among medical students in a large-scale systematic review with a meta-analysis, reported relevant depressive load measured with the PHQ-9 ranging from 4 to 48%.

Among large-scale studies conducted in Germany on the mental health of children, adolescents, and young adults, the BELLA study (i.e., BEfragung zum seeLischen WohLbefinden und VerhAlten) has examined the mental health and health-related quality of life of children and adolescents in Germany in a module within the framework of the Federal Health Reporting at the Robert Koch Institute (35). The DEGS study—that is, the German Health Interview and Examination Survey for Adults—is part of the health-monitoring programme of the Robert Koch Institute and has recorded the national health situation of the adult population in Germany since 2008 (36). Overall, according to the parents' report, ~11% (10%) of children and adolescents had clinically significant symptoms of depression (anxiety). In the self-report, however ~16% (15%) of the youth showed clinically significant symptoms of depression(anxiety). Such heterogeneity of value ranges in reports of psychological stress is well-known. For instance, Erschens et al. (37), who examined some methodological aspects of research on the burden of anxiety and depression among medical students, found that direct comparison and meta-analysis are hindered by the tremendous heterogeneity in the international reporting of prevalence rates of symptoms of anxiety and depression among medical students. A final classification of the results reported here about feelings of depression and anxiety among high school graduates thus remains difficult.

Caspar et al. (38) have mentioned intra- and interpersonal factors involved in the multifactorial causation of symptoms of depression and disorders, in addition to genetics and psychosocial stress. They include, for example, low income, unemployment, low educational attainment, critical life events, and a lack of social support, as well as being a (young) woman, an adolescent, and/or a young adult.

In our study, young women who participated had higher scores for symptoms of depression than young men. No gender-based differences emerged for symptoms of anxiety, however. Results obtained from the survey are consistent with findings in the literature showing that (young) women are more likely to report psychological distress than (young) men, both in the general population and among medical students (13, 35, 36, 39, 40). In the limited number of studies conducted on high school students in general or recently enrolled medical students, a similar trend has been observed (12, 20, 23). Nevertheless, some of their findings have not been significant.

No differences in affective symptoms surfaced between the high school graduates whose parents were employed in a medical profession vs. ones whose parents were not. Past studies have primarily examined the relationship between parents employed in the medical profession and the medical specialization, academic performance, employment as a rural physician, academic satisfaction, and academic performance of medical students (41).

Participants who are solely interested in medicine and not any other subjects tended to have marginally higher levels of symptoms of depression or anxiety than graduates who were also interested in other subjects. Erschens et al. (41) examined a similar cohort regarding the association between perceived stress and specific private and training-related stressors in a cross-sectional design comparing them with already enrolled medical students. Their findings suggest similar values for stress among the graduates as in first-year students. The values were significantly lower than those among students in the third, sixth, ninth, and twelfth semesters. The increase in stress could be related to evidence that exposure to the curriculum increases the distress of medical students (1, 42). The trend toward higher scores for feelings of depression and anxiety among high school graduates interested exclusively in studying medicine could also be related to a possible anticipatory concern about failing the *numerus clausus*, which describes a required Grade Point Average to be considered for enrolment in medical school and is an important factor in the selection procedure for human medicine in Germany. During the 2 years of upper school in which students are in the selection process, they need to consistently achieve exceptionally good grades in order to achieve the required *numerus clausus*. Focusing on only one subject could also increase their intrapersonal stress. New concepts may have to be developed if a curriculum is not received, and perhaps time has to be bridged and new, temporary identities built, including by taking up a medically associated professional activity or even a completely new orientation.

### Coping Styles Among High School Graduates: Questions II and III

Results show that the calculated objective association and subsequent ranking of (dys)functional coping styles, which are associated with a lower (higher) ORs for feelings of depression and anxiety, broadly align with the subjective assessment of the degree to which coping styles are perceived to be helpful. The seven relevant functional coping styles identified for the high school graduates aiming to study medicine were accessing social support from family, accessing social support from friends, accessing social support from by classmates (see Figure 2), relaxing, playing sports, making or listening to music, and reading books. Functional coping styles perform a key role in managing specific distress, as well as in transitioning from critical life stages, including from high school to college and into the earliest stages of medical education (1, 2, 30).

Altogether, few studies have measured coping and the manageability of symptoms of anxiety and depression among high school graduates in general and specifically among those interested in studying medicine. Generally, the students in those studies have already enrolled in medicine or are in their first semester of medical school; therefore, the results presented here on coping styles among high school graduates have to be understood in light of studies on medical students.

In a questionnaire study on coping styles for stress and burnout among medical students in Germany in different phases of their medical education (i.e., freshman, 3rd, 6th, 9th, and 12th semesters), social support, especially from family, friends, and fellow students, emerged as particularly helpful in coping with stress and burnout (30). Moczko et al. (43) also interviewed medical students in Germany in focus groups and found social support to be a highly relevant coping strategy. International studies have also shown that social support is a possible predictor of reduced distress and burnout in medical students and adolescents (44–47). Results on coping styles among premedical high school graduates in the study presented here can be interpreted in relation to literature on medical education internationally, which also considers sports and relaxation methods as helpful styles for reducing stress and protecting against burnout among medical students (48–50). Research on depression has shown how helpful those styles are in preventing and reducing feelings of depression. Thus far, however, there is no definite indication in the literature as to which technique offers the greatest potential for reducing stress among medical students and physicians (6).

The six relevant dysfunctional coping styles identified for the high school graduates were consuming alcohol, abusing drugs, restrictive eating, watching TV, surfing the Internet, and withdrawing or ruminating. The results of a survey of medical students in Germany on dysfunctional coping styles and the prevalence of burnout are consistent with the findings for the premedical high school graduates examined here. Medical students have been shown to engage in alarming coping styles such as increased alcohol consumption and using stimulants and/or sedatives a well as rather passive coping styles such as withdrawing and rumination, and increasing computer activity, all of which demonstrate a higher prevalence of burnout (30). Dyrbye et al. (1) examined different coping styles among U.S. medical students in relation to mental health and found that styles associated with withdrawal such as problem avoidance, social withdrawal, and self-criticism tend to evoke feelings of anxiety, uselessness, incompetence, guilt, and anger as well as impair mental and physical health. Freudenberger (51) has described withdrawal and rumination as constituting a specific phase in his burnout model, which is a pre-phase of depressive feelings, not of a dysfunctional coping strategy.

A highly alarming result in the presented study was that more than 5% of the high school graduates stated that they use drugs to balance their stress and exhaustion, and more than 12% drink alcohol to reduce stress. Those students had a odds ratio of more than 3 times higher of symptoms of depression and anxiety than ones who did not adopt those behaviors. Studies investigating coping strategies of high school graduates in general also report alcohol and/or drug abuse being used as dealing with stress (23, 52). International literature provides heterogeneous results regarding the prevalence of those maladaptive activities in medical students, ranging from 6 to 53% (53). There is no complete understanding of whether, for example, medical students use drugs and alcohol as a coping strategy to reduce stress and strain or whether they use them because of existing pressures such as anxiety, stress, pressure, and tension associated with high school or private problems (7, 54). However, alcohol and drug abuse, or so-called “cognitive doping,” is not a behavior restricted to students interested in medical study (55, 56). In a study by Dietz et al. (57) in Germany, the doping behavior of students from all disciplines was investigated, and the authors found an estimated prevalence from 18.0 to 22.5% for cognitive doping (e.g., stimulants, cocaine, caffeine, or Methylphenidat) using the unrelated question model for the survey.

## Limitations

Overall, the study aimed to fill gaps in knowledge regarding the association between affective symptoms and coping styles used by high school graduates aiming to study medicine. Although the study was conceptually and methodologically well-conceived, some limitations and restrictions warrant attention. For one, the research was conducted as a prospective cohort study; therefore, the causal relationships between functional and dysfunctional coping styles and symptoms of depression and anxiety are explorative, as suggested by the statistical methods used, but cannot be adequately interpreted. For that reason, any associative relationship has to be interpreted accordingly. To further investigate the causal relationships, future longitudinal research with similar cohorts is necessary. In the cross-sectional cohort study, the high school graduates were surveyed with the help of self-assessment instruments, all of which always risk bias. The needs of the students identified with quantitative methods could be supplemented by findings gained via qualitative methods such as individual interviews or focus groups. Beyond that, both comprehensive and individual activities and experiences could be presented in greater detail, and programmes could be designed more effectively.

## Conclusion

The information from our study on the psychological stress of high school graduates and its relationship to coping styles can support considerations for sustainable student health management. Findings could be embedded in three areas: The systematic analysis and health-promoting design of study and job conditions (i.e., relationship prevention), target group-oriented offers for the promotion of individual competences before and around studies, work, and one's own health (i.e., behavioral prevention), and low-threshold and professional counseling and support services for students in difficult situations and/or suffering from mental health problems (i.e., early detection, early treatment, and crisis intervention) (58). Prospective medical students should receive information about those risk factors, as well as triggering and maintenance mechanisms, and be informed about behavior-resistant factors proven to be effective. Functional styles and rituals already implemented should be identified, maintained, and, if necessary, strengthened. Collaborative learning groups, as well as mentoring programmes for (international) students, have also proven to be effective (59). Upcoming research should investigate further factors and dynamics with cross-sectional and longitudinal studies, including the potential influence of personality dimensions such as conscientiousness as being essential to academic performance and a moderator of mental health in medical students (60). Pronounced extraversion and agreeableness facilitate social interactions with others and the establishment and maintenance of interpersonal relationships (61). For medical students, the association between mental health and a high Sense of Coherence, as an essential personal resource relevant to health (62), has been documented (63, 64). Further studies may assess possible relationships and implications regarding altered university admission procedures for the study of human medicine in Germany for the questions investigated here (41).

## Data Availability Statement

The raw data supporting the conclusions of this article will be made available by the authors, without undue reservation.

## Ethics Statement

The studies involving human participants were reviewed and approved by the Ethics Committees of the Faculty of Medicine at University Hospital Tuebingen approved the study (number 053/2014BO1). All participants provided their written informed consent. The patients/participants provided their written informed consent to participate in this study.

## Author Contributions

This study was designed by RE and FJ. AH-W, CN, and SZ gave substantial input to the research question and the updated theoretical framework. RE, TL, and FS acquired the data. RE and TL ran the statistical analysis. RE generated the figures and tables. RE, KG, and FJ interpreted the data. The first draught of the manuscript was written by RE and all authors commented on previous versions of the manuscript. RE drafted the revisions of the manuscript. All authors read and approved the final manuscript.

## Funding

This study was funded by the Federal State of Baden-Württemberg through the Competence Centre for the Prevention of Mental and Psychosomatic Disorders in Work and Educational Settings Baden-Württemberg [AZ42-04HV.MED(13)/3/1]. We acknowledge support by Open Access Publishing Fund of University of Tübingen.

## Conflict of Interest

The authors declare that the research was conducted in the absence of any commercial or financial relationships that could be construed as a potential conflict of interest.

## Publisher's Note

All claims expressed in this article are solely those of the authors and do not necessarily represent those of their affiliated organizations, or those of the publisher, the editors and the reviewers. Any product that may be evaluated in this article, or claim that may be made by its manufacturer, is not guaranteed or endorsed by the publisher.
